# Flavonoid 4′-*O*-Methylkuwanon E from *Morus alba* Induces the Differentiation of THP-1 Human Leukemia Cells

**DOI:** 10.1155/2015/251895

**Published:** 2015-02-09

**Authors:** Peter Kollar, Tomáš Bárta, Stanislava Keltošová, Pavlína Trnová, Veronika Müller Závalová, Karel Šmejkal, Jan Hošek, Radek Fedr, Karel Souček, Aleš Hampl

**Affiliations:** ^1^Department of Human Pharmacology and Toxicology, Faculty of Pharmacy, University of Veterinary and Pharmaceutical Sciences Brno, Palackého Třída 1-3, 612 42 Brno, Czech Republic; ^2^Department of Histology and Embryology, Faculty of Medicine, Masaryk University, Kamenice 3, 625 00 Brno, Czech Republic; ^3^International Clinical Research Center, St. Anne's University Hospital, Pekařská 53, 656 91 Brno, Czech Republic; ^4^Department of Molecular Biology and Pharmaceutical Biotechnology, Faculty of Pharmacy, University of Veterinary and Pharmaceutical Sciences Brno, Palackého Třída 1-3, 612 42 Brno, Czech Republic; ^5^Department of Cytokinetics, Institute of Biophysics, Academy of Sciences of the Czech Republic, Královopolská 135, 612 65 Brno, Czech Republic

## Abstract

*Aims*. In this work we studied cytodifferentiation effects of newly characterized prenyl flavonoid 4′-*O*-methylkuwanon E (4ME) isolated from white mulberry (*Morus alba* L.). *Main Methods*. Cell growth and viability were measured by dye exclusion assay; cell cycle and surface antigen CD11b were monitored by flow cytometry. For the cytodifferentiation of cells the NBT reduction assay was employed. Regulatory proteins were assessed by western blotting. *Key Findings*. 4ME induced dose-dependent growth inhibition of THP-1 cells, which was not accompanied by toxic effect. Inhibition of cells proliferation caused by 4ME was associated with the accumulation in G1 phase and with downregulation of hyperphosphorylated pRb. Treatment with 4ME led to significant induction of NBT-reducing activity of PMA stimulated THP-1 cells and upregulation expression of differentiation-associated surface antigen CD11b. Our results suggest that monocytic differentiation induced by 4ME is connected with up-regulation of p38 kinase activity. *Significance*. Our study provides the first evidence that 4ME induces the differentiation of THP-1 human monocytic leukemia cells and thus is a potential cytodifferentiating anticancer agent.

## 1. Introduction

Specific types of neoplastic diseases, such as acute promyelocytic leukemia (APL), can be treated with a cytodifferentiating approach [1]. The therapy based on an induction of cytodifferentiating programme of malignant cells has shown good efficacy and low level of toxicity, when compared to a cytotoxic agent based treatment. Retinoids, the natural and synthetic derivatives of vitamin A, are known to play a crucial role in cellular and tissue differentiation [2]. All-*trans*-retinoic acid (ATRA) is used in the treatment/chemoprevention of hematologic and other malignancies [3]. However, the clinical use of ATRA is often limited by resistance and toxicity (particularly retinoic acid syndrome) [4]. In general terms, there are two possible strategies for differentiation therapies: (a) the development of ATRA-based pharmacologic combinations that are more powerful and easily tolerated than the individual components [5] or (b) the identification of a novel agent capable of inducing the cytodifferentiation programme in cancer cells [2].

**Figure 1:**
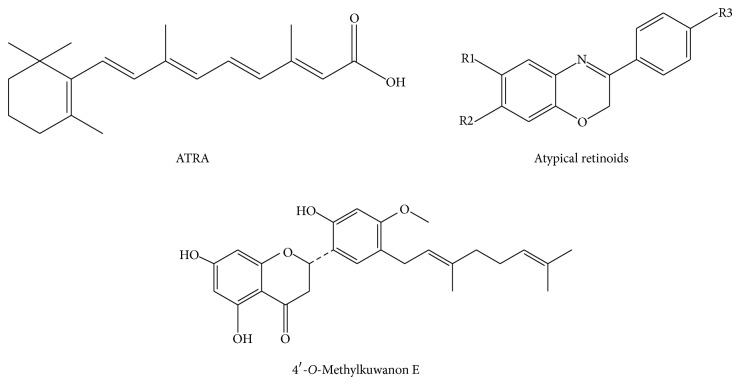
Chemical structures of all-*trans*-retinoic acid (ATRA), atypical retinoids, and 4′-*O*-methylkuwanon E (4ME).

Previously we studied toxicity and biological effects of prenylated and geranylated flavonoids from plants of Moraceae and Paulowniaceae families with cytostatic activity in normal and cancer cell lines [6]. We showed that molecular mechanisms of the antiproliferative effects of geranylated flavanone tomentodiplacone B on human monocytic leukemia cells are mediated through the direct inhibition of CDK2 activity followed by reduced pRb phosphorylation [7]. Flavonoid, cudraflavone B, isolated from* Morus alba* L. (Moraceae) (MA) exerted strong anti-inflammatory properties [8] together with the inhibition of G1/S transition, accompanied by the decreased proliferation and by apoptosis of four cancer cell lines [9]. However, in one of MA isolated flavonoids, 4′-*O*-methylkuwanon E (4ME, the novel compound detected and described in our laboratory), which was tested for its antiproliferative potential, its inhibitory activity on cell cycle could not be explained by significant proapoptotic effect [9]. Thus, we focused our attention on other mechanisms possibly underlying antiproliferative effect of 4ME (Figure 1) on THP-1 leukemia cells. Three different regions can be highlighted in the structure of ATRA: the hydrophobic trimethylhexene ring, the unsaturated linking chain, and the relatively hydrophilic moiety of the carboxylic acid. The basic structure of the atypical retinoids generated through modifications of ATRA using structure-based chemical design strategies is also shown [10]. From structure comparison clear similarities with the chemical formula of flavonoid 4ME can be found. Geranylated (prenylated) flavonoids are compounds usually showing two different structural regions: a lipophilic side chain and relatively hydrophilic ring B, connected to side chain* via* heterocyclic moiety. This general arrangement shows similarities with retinoids and gives to this type of flavonoids interesting properties affecting biological activity [11].

As a molecular structure of 4ME has a certain similarity with atypical retinoids [10] we hypothesized the ability of 4ME to induce differentiation programme in leukemia cells. Therefore, the present study has attempted to investigate the cytodifferentiation effect of prenylated flavonoid 4ME isolated from* M. alba* L. on THP-1 cells.

## 2. Materials and Methods

### 2.1. Test Compounds and Reagents

4ME was isolated and supplied by the Department of Natural Drugs, Faculty of Pharmacy, University of Veterinary and Pharmaceutical Sciences Brno, Czech Republic. The identification of substance was carried out using HRMS, ^1^H and ^13^C NMR analysis; and the purity exceeded 95% according to the HPLC analysis [6]. The compound is poorly soluble in water; therefore, a fresh 10 mM stock solution in dimethyl sulfoxide (DMSO) (Sigma-Aldrich, St. Louis, MO, USA) was prepared every time 1 day prior to experiments and stored at –20°C. This solution was further diluted in the culture media to the desired final concentrations. RPMI 1640 culture media, phosphate buffered saline (PBS), and antibiotics (penicillin and streptomycin) were purchased from Lonza (Verviers, Belgium). Foetal bovine serum (FBS) was purchased from PAA Laboratories (Pasching, Austria). Rabbit polyclonal antibodies against p38 MAPK [pT180/Y182] (9215S) and p38 MAPK (9212) were purchased from Cell Signaling Technologies (Beverly, MA, USA). Mouse monoclonal antibodies against pRb (554136) were purchased from BD Biosciences (Franklin Lakes, NJ, USA). Rabbit polyclonal antibody against phospho-Rb [Ser 780] (9307) was purchased from Cell Signaling Technologies (Beverly, MA, USA). PE-conjugated CD11b antibody was obtained from Beckman Coulter (Brea, CA, USA). ATRA and all other reagents were from Sigma-Aldrich.

### 2.2. Cell Culture

The human monocytic leukemia THP-1 cell line was purchased from the European Collection of Cell Cultures (Salisbury, UK; methods of characterization: DNA fingerprinting (multilocus probes) and isoenzyme analysis). Cells were cultured in RPMI 1640 medium supplemented with antibiotics (100 U/mL penicillin, 100 mg/mL streptomycin), 10% FBS, and 2 mM L-glutamine. Cultures were kept in an incubator at 37°C in a water-saturated 5% CO_2_ atmosphere in air. Cells were passaged at approximately 1-week intervals. Cells were free from mycoplasma infection (Hoechst 33258 staining method).

### 2.3. In Vitro Analysis of Cell Growth and Viability

THP-1 cells were seeded (2 × 10^5^ cells/mL) and incubated for 96 h at 37°C with 5% CO_2_ with 4ME dissolved in DMSO (Sigma-Aldrich) in concentrations ranging from 5 to 20 *μ*M in RPMI 1640 medium. The maximum concentration of DMSO in the assays never exceeded 0.1%. At the indicated time points (24 h, 72 h, and 96 h) cell population in each well was removed by gentle scraping with a cell scraper and harvested for further analysis. Numbers and viabilities of the cells were determined by counting with a hemocytometer as we previously described [7]. All data were evaluated using GraphPad Prism 5.00 software (GraphPad Software, San Diego, CA, USA, http://www.graphpad.com/).

### 2.4. Cell Cycle Analysis

THP-1 cells were incubated with increasing concentrations of 4ME for 24 h, washed in PBS (pH 7.4), and fixed for 30 min in an ice-cold 70% ethanol. Fixed cells were washed three times in PBS (pH 7.4) and incubated with RNaseA (0.02 mg/mL) (Boehringer, Ingelheim, Germany) for 30 min at 37°C. Nuclei were stained with propidium iodide (40 *µ*g/mL) and analysed by flow cytometry using Cell Lab Quanta SC (Beckman Coulter, Brea, CA, USA). Cell cycle distribution was analysed using FlowJo software (http://www.flowjo.com/).

### 2.5. In Vitro Analysis of Cytodifferentiation

Functional assays of differentiation were based on the ability of phorbol myristate acetate-stimulated human monocytic leukemia cells to reduce nitroblue tetrazolium (NBT). Samples of 5 × 10^5^ cells were incubated in 0.5 mL of RPMI 1640 with penicillin, streptomycin, glutamine, and 10% heat-inactivated FBS containing 0.25 mg/mL of NBT (Roche Applied Science, Mannheim, Germany) and 500 ng/mL phorbol-12-myristate-13-acetate (PMA). Cells were incubated at 37°C for 25 min after which the samples were centrifuged (3 000 g for 5 minutes) and 1% Triton X-100 (Sigma-Aldrich) was added in the amount of 1 mL/5 × 10^5^ cells. Samples were sonicated (Sonicator S-3000, Misonix Inc., Farmingdale, USA) and intensity of dark-blue formazan was assessed by spectrophotometry analysis at OD 540 nm. For determination of the number of adherent cells THP-1 cells (2 × 10^5^ cells/mL) were placed into 6-well tissue plate and cultured for 72 and 96 h. Nonadherent cells were removed by washing twice with PBS and then adherent cells were collected by gentle scraping with a cell scraper and by vigorous pipetting. The number of cells was counted by a hemocytometer. Morphological changes of THP-1 cells were detected using an inverted microscope (Axiovert 40 CFL, Zeiss, Germany).

### 2.6. Western Blotting

Cells were washed three times with PBS (pH 7.4) and lysed in 100 mM Tris-HCl (pH 6.8) containing 20% glycerol and 1% SDS. Protein concentrations were determined using the DC Protein Assay Kit (Bio-Rad, Hercules, CA, USA). Lysates were supplemented with bromophenol blue (0.01%) and *β*-mercaptoethanol (1%). Equal amounts of total protein were separated by SDS-polyacrylamide gel electrophoresis (PAGE), electrotransferred onto PVDF membranes (Millipore, Billerica, MA, USA), immunodetected using the appropriate primary and secondary antibodies, and visualised with ECL Plus reagent (Amersham, Aylesbury, UK) according to the manufacturer's instructions.

### 2.7. Analysis of Surface Differentiation Marker CD11b by Flow Cytometry

THP-1 cells were plated at 3 × 10^5^ cells/mL, 2 mL/well (6 × 10^5^ cells/2 mL) in 6-well plate and incubated with ATRA (1 *μ*M), 4ME (5, 10, and 20 *μ*M, resp.), or RPMI 1640 only for 72 or 96 h in duplicate for each sample. A number of 5 × 10^5^ cells from each well were collected at the indicated time points (72 h, 96 h), centrifuged (1.300 rpm for 3 min), washed twice with PBS, and incubated with PE-conjugated CD11b antibody (20 *μ*L/5 × 10^5^ cells, Beckman Coulter) for 30 min at room temperature in the dark. After incubation the cells were washed with PBS, suspended in PBS, and analysed by flow cytometry using Cell Lab Quanta SC flow cytometer and Kaluza Analysis Software (Beckman Coulter).

### 2.8. Statistical Analysis

Statistical significance was tested using the one-way ANOVA with Dunnett's and Tukey post test for comparisons between the mean values, and differences between two conditions were retained for *P* < 0.05. Statistical significance was determined at levels of *P* < 0.05, *P* < 0.01, and *P* < 0.001.

## 3. Results

### 3.1. Growth Inhibitory Effects and Morphological Changes Induced by 4ME to THP-1 Cells

**Figure 2:**
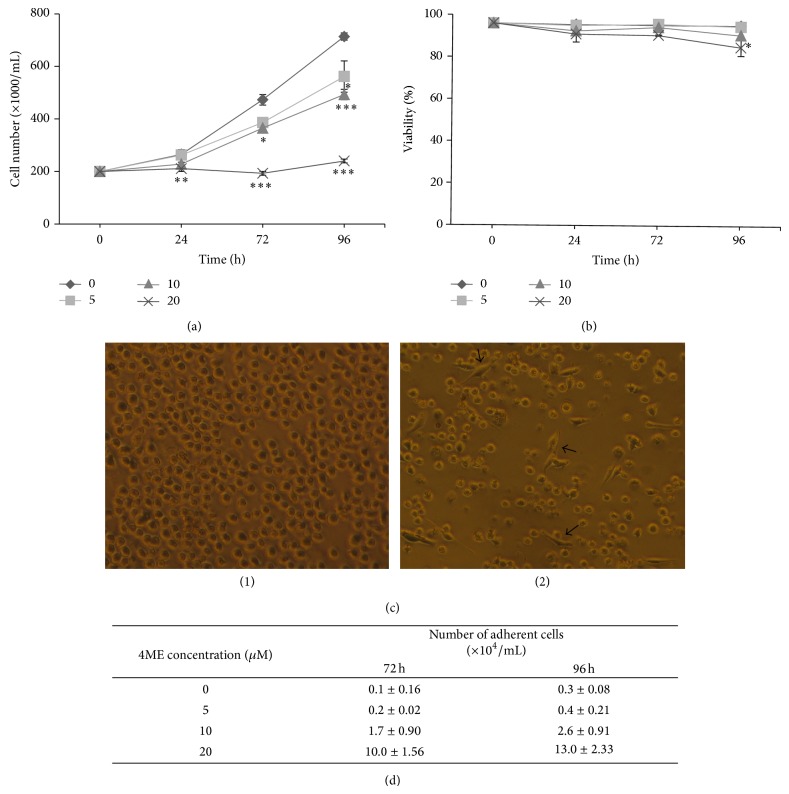
Growth inhibition and influence on viability and morphological changes of THP-1 cells by 4ME. Cells were seeded (2 × 10^5^ cells/mL) and cultured with 0 (◆), 5 (■), 10 (▲), and 20 (×) *µ*M 4ME for 96 h. Aliquots of cells were used for the determination of total number of cells (a) and cell viability (b). The results shown are expressed as the mean ± SD of two independent experiments, with each condition tested in triplicate. ^*^ *P* < 0.05; ^**^ *P* < 0.01; ^***^ *P* < 0.001, significantly different from control. (c) Morphological changes of THP-1 monocytic leukemia cells after 72 h treatment with 4ME. (1) Vehicle treated THP-1 cells and (2) 4ME (20 *µ*M) treated THP-1 cells. In comparison with monocytes, differentiated macrophages tend to adhere to the bottoms of the cultivation plates, as indicated with arrows (50x magnification). (d) THP-1 cells (2 × 10^5^ cells/mL) were cultured for 72 and 96 h. The number of adherent cells was determined as described in Materials and Methods. Each value represents the mean ± SD.

To investigate how 4ME influences cell proliferation we examined its growth-inhibiting effects on THP-1 cells at three time points (after 24, 72, and 96 h, resp.). Simultaneously, we evaluated viability of cells upon 4ME treatment. Antiproliferative effect of 4ME on THP-1 cells was found to be time- and concentration-dependent, the most significantly observed (*P* < 0.001) at concentrations of 10 and 20 *µ*M (Figure 2(a)). After 72 h, 4ME treatment of THP-1 cells leads to significant growth inhibition of THP-1 cells when concentrations of 10 *µ*M (*P* < 0.05) and 20 *µ*M (*P* < 0.001) were used. The lowest concentration of 4ME (5 *µ*M) used was effective (*P* < 0.05) after 96 h of the treatment. No toxic effect of 4ME has been detected throughout the evaluation time as viability was not significantly changed, except the highest 4ME concentration at 96 h (*P* < 0.05) with 85% viability, which cannot be considered as toxic (Figure 2(b)). 4ME also induced the morphologic changes of THP-1 cells into macrophage-like cells with different cell shape and ability to attach the surface of plastic culture dishes (Figure 2(c)). The number of adherent cells after 72 and 96 h of cultivation increased in the presence of 4ME in a dose-dependent manner (Figure 2(d)). This effect was significant from 10 *µ*M of the drug at both time points.

### 3.2. Effects of 4ME on Distribution of Cells in Cell Cycle Phases

**Figure 3:**
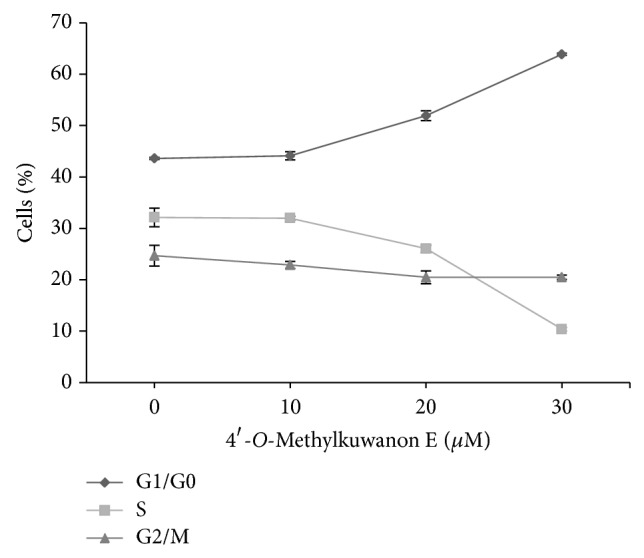
Treatment with 4ME causes accumulation of human monocytic leukemia THP-1 cells in G1/G0 phase. Cell cycle distribution at 24 h upon treatment of THP-1 cells with 4ME as determined by flow cytometry. Values shown are the mean ± SEM of the percentages of cells in individual phases of the cell cycle from two independent experiments.

Antiproliferative effect of anticancer drugs is usually accompanied by significant changes of the cell cycle. To determine which changes in the cell cycle occur after 4ME treatment of THP-1 cells we performed cell cycle analysis based on DNA content using flow cytometry. As shown in Figure 3 4ME caused the accumulation of human leukemia cells in G1/G0 phase dose-dependently after 24 h treatment. While the percentage of S phase cells decreased, the percentage of cells in G2/M phase remained unchanged upon 4ME treatment.

### 3.3. Effect of 4ME on NBT-Reducing Activity

**Figure 4:**
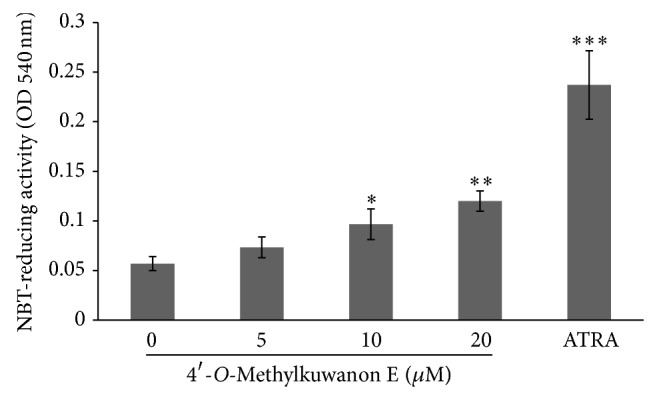
4ME causes increased NBT reduction in PMA stimulated THP-1 cells. Cells were cultured with indicated concentrations of 4ME or 1 *µ*M ATRA, added as a positive control, for 72 h. Aliquots of cells were used for the determination of NBT-reducing activity. The results shown are expressed as the mean ± SD of three independent experiments, with each condition tested in triplicate. ^*^ *P* < 0.05; ^**^ *P* < 0.01; ^***^ *P* < 0.001, significantly different from control.

Reduction of NBT is considered to be a typical marker of myelomonocytic differentiation in leukemia cells [12]. To investigate whether 4ME triggers the cytodifferentiating programme in monocytic leukemia cells, we have employed NBT reduction assay. Results showed that 4ME significantly induced NBT reduction in monocytes after 72 h in a dose-dependent manner (Figure 4). In concentration of 20 *µ*M 4ME caused more than twofold higher (*P* < 0.01) induction of NBT reduction in comparison with the vehicle-treated cells. The NBT-reducing activity of THP-1 cells was also induced by 1 *µ*M ATRA, added as a positive control.

### 3.4. Expression of Differentiation-Associated Cell Cycle Regulators in 4ME-Treated Cells

**Figure 5:**
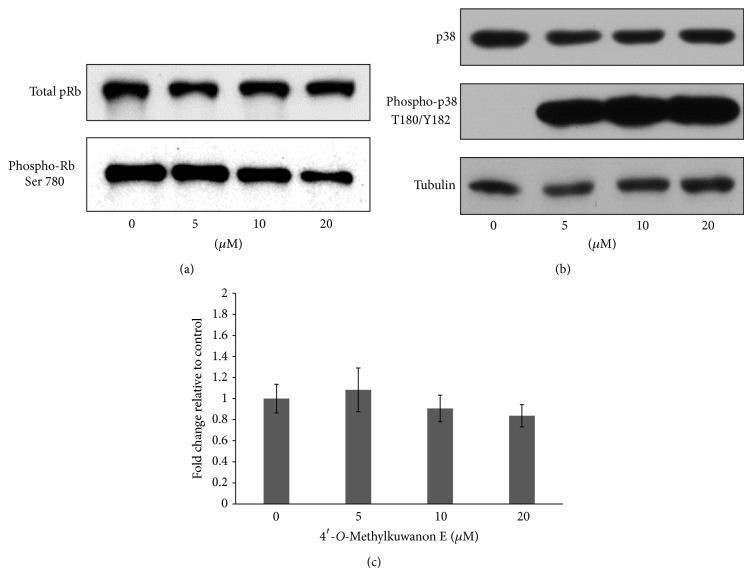
Expression of differentiation-associated cell cycle regulators after 72 h of 4ME treatment. (a) pRb phosphorylation on Ser 780 was reduced after 72 h of 4ME treatment, as determined by western blot analysis. (b) Following 72 h of challenge, 4ME increases p38 phosphorylation in all concentrations used. Data representative of two other experiments are shown. Panel (c) shows quantification of p38 expression at protein level in THP-1 cells after 72 h of 4ME treatment as determined by western blot analysis. Values shown are mean ± SD of two independent experiments performed in duplicate.

Based on the findings that 4ME causes growth inhibition, accumulation of cells in G1/G0 phase, and induction of NBT reduction in THP-1 cells we determined the expression and phosphorylation status of key cell cycle proteins involved in monocytes differentiation. The retinoblastoma protein (pRb) is currently known to have a prominent role in control of cellular proliferation and differentiation. Rb dephosphorylation leading to inhibition of G1/S transition is a prerequisite for initiating the process of cytodifferentiation. The proportion of hyperphosphorylated (phosphoserine 780) pRb was markedly reduced dose-dependently in THP-1 cells exposed for 72 h to 4ME treatment (Figure 5(a)). Since it has been known that the p38 signalling pathway plays a significant role in the differentiation process [13], THP-1 cells were cultivated with various concentrations of 4ME for 72 h and subjected to immunoblot analysis with anti-phospho-p38. As shown in Figure 5(b), the phosphorylation of p38 was upregulated by 4ME treatment at all concentrations used, suggesting that this kinase could be involved in 4ME-induced cytodifferentiation.

### 3.5. Effect of 4ME on Expression of Differentiation-Associated Surface Antigen CD11b

**Figure 6:**
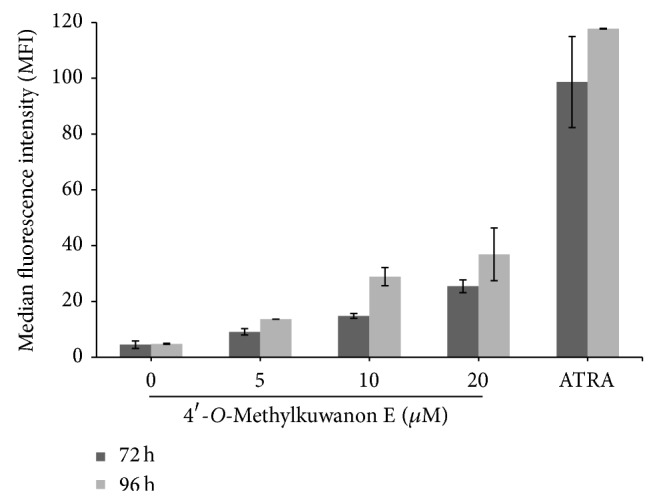
Increased expression of differentiation-associated surface antigen CD11b in 4ME-treated cells. THP-1 cells were cultured with indicated concentrations of 4ME or 1 *µ*M ATRA, added as a positive control for 72 and 96 h. The level of expression of the indicated phenotypic marker was determined by flow cytometry using fluorescein-labelled monoclonal antibody against CD11b. The amount of cell-associated fluorescence in median fluorescence intensity (MFI) is illustrated in the graph. The results shown are expressed as the mean ± SD of three independent experiments, with each condition tested in duplicate.

Monocytic differentiation is associated with increased expression of CD11b/CD18 [14]. Flow cytometric determination of THP-1 cells surface levels of the CD11b has been used as an index of 4ME-induced activation of these cells to macrophages. Monocytes were treated for 96 h with increasing 4ME concentrations or with 1 *µ*M ATRA. The expression of CD11b, a cell surface marker of macrophage-like differentiation, was increased time- and dose-dependently by 4ME after both 72 and 96 h (Figure 6). The strongest effect was observed in ATRA-treated cells; still, the amount of cell-associated fluorescence (MFI) after 4ME treatment was more than fivefold higher when compared to control.

## 4. Discussion

Differentiation therapy is conceptually an elegant approach to the eradication of neoplastic cells from the human body because cytotoxicity is avoided, whereas normal mature cells are unaffected by the differentiation agents [15]. The most extensively studied differentiation agents in cancer medicine include ATRA, 9-*cis*-retinoic acid, and 13-*cis*-retinoic acid. ATRA is one of the most biologically active retinoids, and several clinical studies have established that ATRA can induce differentiation of leukemia cells and remission [16]. However, the occurring resistance and toxicity of ATRA therapy underline the importance of searching for new compounds capable to switch the differentiation programme in leukemia cells.

In this work we studied cytodifferentiation effects of newly characterized prenyl flavonoid 4ME isolated from white mulberry (*Morus alba* L.). Our previous results [9], some structural similarities with atypical retinoids, and unpublished observations turned our attention to mechanisms different from direct toxic effect, stress-related or apoptotic signaling pathways, which might be involved in the action of this compound. We found that 4ME was able to induce dose-dependent growth inhibition of THP-1 cells, which was not accompanied by toxic effect (Figures 2(a) and 2(b)). Moreover, with 4ME added, the THP-1 cells morphologically resembled macrophages (Figure 2(c)) and increased the number of adherent cells (Figure 2(d)). These findings together with accumulation in G1 phase found by flow cytometry (Figure 3) prompt us to gain more detailed insight into the mechanism of 4ME action. The crucial process in terminal differentiation is to delay proliferation in G1/G0 phase of the cell cycle and thus establish somatic cell cycle programme. pRb is the key player involved in G1 phase regulation and its phosphorylation, which is required for G1/S transition was significantly inhibited by 4ME treatment (Figure 5(a)). We assume that this allowed THP-1 monocytes to escape from the cell cycle machinery and subsequently to initiate cytodifferentiation. Indeed, three-day treatment with 4ME led to significant induction of NBT-reducing activity following priming of the cells with PMA, accompanied by upregulated expression of differentiation-associated surface antigen CD11b (Figures 4 and 6). Although these effects are lower than effects of ATRA still cytodifferentiation induced by 4ME could be considered as clearly evident. Since p38 pathway has been indicated to be involved in the differentiation of several human cells [13], we have focused on p38. Its activation can lead to biological outcomes such as proliferation, cell survival, and differentiation, depending on the context and the cell type [17]. As it is shown in Figure 5(c) after 72 h treatment with 4ME the expression of p38 protein was not enhanced, while its phosphorylated form was significantly increased (Figure 5(b)). These results suggest that monocytic differentiation induced by 4ME is associated with upregulation of p38 kinase activity. Still, the connection between differentiation and this signaling route remains to be elucidated.

In conclusion, this is the first study to demonstrate cytodifferentiating activity of prenylated flavanone 4ME in human monocytic leukemia cells. This promising effect was induced at nontoxic concentrations. However, our findings using THP-1 cell line do not completely reflect the situation in patients with acute leukemia. Further experiments using mice models inoculated with L1210 mice leukemia cells or freshly isolated primary human leukemic cells are necessary to verify cytodifferentiation effect of 4ME.

## 5. Conclusion

The present study has shown that 4ME induced dose-dependent growth inhibition of THP-1 cells, which was not accompanied by toxic effect. Inhibition of cells proliferation caused by 4ME was associated with the accumulation in G1 phase and with downregulation of hyperphosphorylated pRb. Treatment with 4ME led to significant induction of NBT-reducing activity of PMA stimulated THP-1 cells and upregulated expression of differentiation-associated surface antigen CD11b. Our results suggest that monocytic differentiation induced by 4ME is connected with upregulation of p38 kinase activity.

## Abbreviations

4ME: 4′-*O*-Methylkuwanon E
APL: Acute promyelocytic leukemia
ATRA: All-*trans*-retinoic acid
DMSO: Dimethylsulphoxide
FBS: Foetal bovine serum
NBT: Nitroblue tetrazolium
PBS: Phosphate buffered saline
PMA: Phorbol-12-myristate-13-acetate
pRb: Retinoblastoma protein
THP-1: Human monocyte leukemia cell line.
